# Epilepsy and Other Chronic Convulsive Diseases, Etc.

**Published:** 1886-04

**Authors:** 


EPILEPSY AND OTHER CHRONIC CONVULSIVE DISEASES; THEIR CAUSES, SYMPTOMS AND TREATMENT. *By* W. R. GOWERS, M.D., F.R.C.P. *Large octavo, pp. xi, 255. New York:* WILLIAM WOOD & CO. *Chicago:* W. T. KEENER.

This work is largely based on the carefully recorded histories of some 1,450 cases, many of them having been treated in The National Home for the Paralyzed and Epileptic. This mass of clinical material has been carefully worked up, and the result is an exceedingly valuable monograph. The author does not make the too common mistake of tabulating all his figures, and allowing the reader to draw his conclusions from the footings of each item, but he has made a thorough analysis, and in the main the conclusions are just and accurate.

The diagnosis ot "hysteroid" attacks is very clearly explained, as well as their relation to other convulsive diseases.

The chapter on treatment is perhaps the most unsatisfactory in the whole book, and contains very little that is new. Until the intimate pathology of epilepsy shall be known, when some safe generalizations may be made concerning the action of drugs in this disease, treatment will probably continue to be unsatisfactory. The author wisely, we think, refrains from any deductions of this kind. The references to the literature of the subject are somewhat scanty, but those that are given cover a wide range. We think an extensive bibliography would have been more in keeping with the character of the work. It is surprising to find that the publishers have bound sixty-four pages ot advertising matter in the back of such a book as this, but even that will not prevent many from purchasing Dr. Gowers' really valuable work.

